# AI-CenterNet CXR: An artificial intelligence (AI) enabled system for localization and classification of chest X-ray disease

**DOI:** 10.3389/fmed.2022.955765

**Published:** 2022-08-30

**Authors:** Saleh Albahli, Tahira Nazir

**Affiliations:** ^1^Department of Information Technology, College of Computer, Qassim University, Buraydah, Saudi Arabia; ^2^Faculty of Computing, Riphah International University, Islamabad, Pakistan

**Keywords:** DenseNet, localization, CenterNet, chest X-ray images, deep learning

## Abstract

Machine learning techniques have lately attracted a lot of attention for their potential to execute expert-level clinical tasks, notably in the area of medical image analysis. Chest radiography is one of the most often utilized diagnostic imaging modalities in medical practice, and it necessitates timely coverage regarding the presence of probable abnormalities and disease diagnoses in the images. Computer-aided solutions for the identification of chest illness using chest radiography are being developed in medical imaging research. However, accurate localization and categorization of specific disorders in chest X-ray images is still a challenging problem due to the complex nature of radiographs, presence of different distortions, high inter-class similarities, and intra-class variations in abnormalities. In this work, we have presented an Artificial Intelligence (AI)-enabled fully automated approach using an end-to-end deep learning technique to improve the accuracy of thoracic illness diagnosis. We proposed AI-CenterNet CXR, a customized CenterNet model with an improved feature extraction network for the recognition of multi-label chest diseases. The enhanced backbone computes deep key points that improve the abnormality localization accuracy and, thus, overall disease classification performance. Moreover, the proposed architecture is lightweight and computationally efficient in comparison to the original CenterNet model. We have performed extensive experimentation to validate the effectiveness of the proposed technique using the National Institutes of Health (NIH) Chest X-ray dataset. Our method achieved an overall Area Under the Curve (AUC) of 0.888 and an average IOU of 0.801 to detect and classify the eight types of chest abnormalities. Both the qualitative and quantitative findings reveal that the suggested approach outperforms the existing methods, indicating the efficacy of our approach.

## Introduction

The easier availability of multimedia content such as digital images and videos has enhanced the growth of tasks performed in the field of computer vision (CV). The well-known applications of CV involve object detection (1), object tracking (2), medical image analysis (3–5), text analysis (6, 7), and video processing (8). The usage of CV approaches in the area of medical image analysis is assisting the practitioners to perform their jobs quickly and accurately. One of such applications is chest X-ray (CXR) analysis. The CXR is the highest employed modality in the world to identify several thoracic abnormalities such as pneumonia, COVID-19, atelectasis, and lung nodule. The easier and more economic behavior of CXR leads to significant medical inspections every day (9). However, the manual examination of CXR is highly reliant on the availability of domain specialists. Moreover, the manual CXR study is a taunting and time-taking activity accompanying high chances of wrong predictions. Whereas, the automated CXR recognition system can fasten this process and increase the accuracy of the system as well.

Chest abnormalities are the major reasons of deaths and disability around the globe with about 65 million people suffering from one disease or the other and 3 million demises per year. Hence, timely identification of such diseases can save the lives of patients and protect them from painful treatment procedures (10). Therefore, to tackle the problems of manual CXR inspection, the researchers have focused their attention to present reliable automated solutions. Initially, the handcrafted feature computation approaches were used for the classification of several CXR abnormalities. Such methods are simple in nature and can work well-with a small amount of data (11, 12). However, the handcrafted key points calculation methods need extensive domain information and take huge time to produce accurate results. Furthermore, there remains always a trade-off between time complexity and classification results for such techniques. The employment of huge key points enhances the recognition power of these methods but at the cost of the increased computational burden (12). The usage of small key points causes increase in the efficiency of the hand-coded approaches but results in missing acquiring the significant aspect of image modality which in turn decreases the classification results. Due to such reasons, these methods are not found to be proficient for the CXR analysis (13).

Now, the success of Artificial Intelligence (AI)-based techniques in the automatic diagnosis of medical diseases is astonishing. AI, when applied to the medical field, helps with managing, diagnosing, and treating patients. This reduces the stress of physicians and also serves as a helping hand to them. It also helps on the administrative side by automating and managing a large portion of the administrative burden (14). Recently, the advancement of deep learning (DL) frameworks is attracting the attention of the research community to use them for digital image processing including the CXR examination (15, 16). Numerous well-explored DL models such as CNN (17) and Recurrent neural networks (RNNs) (18) are used for segmentation and classification problems. This makes deep learning a very powerful tool in healthcare, as most of the work being done is categorized as either a classification or a segmentation task. The empowerment of DL approaches has made them highly suitable for medical image analysis as these frameworks are capable of computing a more discriminative set of key point vectors without the need for area specialists. The CNN models are inspired by the working of human brains to visualize and recall several objects. The well-known CNN models i.e., VGG (19), ResNet (20), DenseNet (21), and EfficientNet (22) are highly used for several image classification tasks. Such methods can exhibit reliable performance with minimum processing time (23–25). The main idea of using the DL-based techniques for the medical image examination is that these approaches are capable of computing the fundamental information of the input samples and can deal with complex image distortions such as intensity and color variations, noise, blurring, and size changes.

Although existing techniques have acquired inspiring CXR classification results; however, there is space for enhancement both in terms of computational complexity and classification accuracy. Hence, a more comprehensive investigation of the existing traditional machine learning (ML) and DL frameworks is required that can increase the CXR-related disease classification performance. The major problem of ML methods for the CXR abnormality classification is their low effectiveness with increased computational time (26). The power of DL approaches to resolve complicated real-world issues is remarkable in comparison to human brain intellect. While the DL approach resolves problems of ML techniques, however, increased the model complexity as well. Hence, there is a need for a more robust approach to the CXR-related disease classification.

The timely and accurate classification of several CXR diseases is a complex job due to the extensive similarities found among different chest abnormalities. Besides, the incidence of noise, blurring, light variation, and intensity changes in the input samples further complicates the classification procedure. To tackle the problems of existing methods, we have presented a novel framework namely AI-CenterNet CXR to detect and classify eight types of chest abnormalities. More clearly, we have presented the DenseNet-41-based CenterNet approach, where the key points from the input samples are computed by using the DenseNet-41 model. The computed features are later localized and classified by the one-stage object detector of the CenterNet model. The experimental results show that our technique is capable of discriminating various types of chest diseases effectively under the presence of different image distortions. The key contributions of our work are:

- We proposed a novel AI-enabled framework namely AI-CenterNet CXR with DenseNet-41 as a feature extractor to enhance the identification and classification results of eight types of chest abnormalities.
- The presented method is capable of accurately locating and classifying the diseased portion from the X-ray samples because of the effectiveness of the CenterNet technique.
- We have improved the classification performance because of the ability of the AI-CenterNet CXR model to better deal with the model's over-tuned training data.
- We have presented a computationally robust model to classify several CXR abnormalities due to the one-stage object detector framework of CenterNet.
- Huge evaluation is presented, and extensive experimentation is performed against the latest approaches for the CXR disease classification on a complex dataset namely NIH Chest X-ray to show the accurateness of our approach.

## Related work

A lot of research work is proposed in the area of CXR disease detection. This section provided a brief review of previous research done for the detection of multi-class chest diseases from medical images. Ayan and Ünver (27) proposed a DL-based method using Xception and Vgg16 CNN models for the diagnosis of pneumonia. Initially, different data augmentation techniques, such as rotation, zooming, and flipping, were applied to the input images to increase diversity and avoid overfitting. Then, the DL models were fine-tuned using transfer learning to extract discriminative key points. The results showed that the Xception network achieved better classification accuracy as compared to Vgg16; however, the performance can be further improved. Bhandary et al. (28) suggested a DL-based framework for the identification of pneumonia and lung cancer that included two different models. The first network was based on a modified AlexNet (MAN) model to identify pneumonia class. The second network was built using an ensemble strategy that combined handcrafted features collected by the Haralick and Hu approach (29) with deep features from the MAN model. For classification, the Support Vector Machine (SVM) classifier was employed and its performance was compared with the softmax classifier. This technique attained a classification accuracy of 97.27% using CT images from LIDC-IDRI benchmark dataset. In (30), the authors evaluated the performance of different pre-trained CNN models such as GoogLeNet, InceptionNet, and ResNet using different image sizes and transfer learning. Moreover, the network visualization was used to analyze the features learned by these models. The results showed that shallow networks, such as GoogleNet, outperform deeper network architectures for discriminating between healthy and abnormal chest X-rays. Rajpurkar et al. (31) presented a DL-based model namely CheXNet to identify different illnesses in chest. The model was comprised of 121 layers utilizing dense connectivity and batch normalization. The authors retrained the ChexNet model, which had previously been trained on ImageNet data, using the CXR dataset. This approach achieved an F1 score of 43.5% and Area Under the Receiver Operating Characteristic curve (AUROC) of 0.801. In (32), the author proposed a DL model for COVID-19 illness categorization across a wide range of other chest diseases (multi-class classification) from chest x-rays. They employed a Generative Adversarial Networks (GAN)-based approach to generate synthetic images to solve the issues of class imbalance data. The author analyzed the performance using various scenarios such as data augmentation, transfer learning, and imbalanced class data. The results showed that the ResNet-based model yields higher accuracy of 87% with balanced data. Ho and Gwak (33) designed a two-stage approach for the precise identification of 14 different diseases from chest x-ray images. Initially, the abnormal region was localized using activation weights obtained from the last convolutional layer of fine-tuned DenseNet-121 network. Then, classification was performed by using a combination of handcrafted feature extractors i.e., SIFT, HOG, LBP, GIST, and deep features. Several supervised learning classifiers such as SVM, KNN, AdaBoost, and others were used to classify hybrid features. The experimental findings showed that the Extreme Learning Machines (ELM) classifier performs well in comparison to other classifiers, with an accuracy of 0.8462. In (34), the authors developed a CNN-based network comprising three convolutional layers for the identification of 12 different diseases using the CXR samples. They investigated the performance against competitive NN and backpropagation NN with unsupervised learning. The results demonstrated that the proposed CNN attains high recognition rates and better generalization power due to robust feature learning. However, computation time and convergence iterations were slightly higher.

In (35), the authors designed a multi-scale attention network for enhanced multi-class chest disease identification accuracy. The proposed network employed DenseNet169 as a backbone with a multi-scale attention block that fused local characteristics gathered at different scales with global features. A novel loss function using perceptual and multi-label balance was also introduced to solve issues of data imbalance. This approach achieves an AUROC of 0.850 on CheXpert and 0.815 on the CXR dataset. Ma et al. (36) suggested a cross-attention-based, end-to-end architecture to address class unbalanced multi-label x-ray chest illness classification. The model comprised a feature extraction network based on densenet121 and densenet169 as its backbone and a loss function based on an attention loss and multi-label balance loss for better key point representation through mutual attention. This model showed an improved AUROC of 0.817 on the Chest X-ray14 dataset. Wang and Xia (37) presented the ChestNet model to improve the accuracy of multi-class thoracic illness diagnosis using chest radiography. The model was comprised of two sub-networks: classification and attention network. The classification network was based on a pre-trained ResNet-152 model that was used to extract unified key points. The attention network was used to investigate the relationship between class labels and abnormal regions by using the extracted key points. The suggested model outperformed the existing models in classification using the CXR dataset. Ouyang et al. (38) presented an approach to simultaneously perform both abnormality localization and multi-label chest disease classification. The model was based on the hierarchical visual attention mechanism comprising three levels and was trained using a weakly supervised learning algorithm due to the limited number of available box annotations for the abnormal region. This approach exhibited a mean AUC score of 0.819 over the CXR dataset.

Pan et al. (39) used pre-trained DenseNet and MobileNetV2 models for categorizing chest radiographs as healthy or diseased. They evaluated these models for 14 different chest pathologies. To analyze the generalization ability, the authors utilized two different datasets. The results showed that MobileNetV2 outperformed the DenseNet model in the majority of scenarios. Albahli and Yar (40) presented a multilevel classification approach using DL to diagnose COVID-19 and other chest disorders using CXR images. Initially, the first model was used to classify the input into three classes: normal, COVID-19 affected, and other. The second model was then used to perform classification into 14 chest and associated disorders. The suggested approach was evaluated using different pre-trained DL models such as ResNet50, NasNetLarge, Xception, InceptionV3, and InceptionResNetV2. The results exhibit that ResNet50 performed best with an average accuracy of 71.905% for COVID-19 identification and 66.634% for other diseases. Alqudah et al. (41) introduced an approach for the diagnosis of bacterial and viral pneumonia from healthy chest radiographs. Initially, a modified CNN model pre-trained on other medical images was fine-tuned to learn pneumonia disease-specific features. Then, classification was performed using different classifiers such as softmax classifier, SVM, and KNN. The results exhibit that SVM outperformed the other classifiers; however, the performance was evaluated on the limited dataset. Kim et al. (42) presented an end-to-end learning approach to perform multi-label lung disease classification. Initially, the input images were preprocessed by applying crop and resize operations to remove meaningless information from images. Then, the pre-trained EfficientNetv2 model was fine-tuned using input images for the extraction of discriminative key point vector and then classified into respective classes. This method depicts improved results for three-class classification; however, the model suffers from overfitting and performance degrades on increasing the number of classes. Baltruschat et al. (43) examined the execution of various ResNet-based models for the task of multi-label chest x-ray images. The authors extended the architecture and incorporated non-image features such as the patient's age, gender, and image acquisition category in the network for improved classification. The results show that ResNet-38 with integrated meta-information performed best with an AUC of 0.727 as compared to others. Ibrahim et al. (44) presented a DL-based multi-class identification method using both CXR and CT images. The authors compared four different custom architectures based on VGG19, ResNet152V2, and Gated Recurrent Unit (GRU). The results exhibit that custom VGG-19 outperformed the other models (i.e., ResNet152V2, ResNet152V2 followed by GRU and Bi-GRU) by attaining an accuracy of 98.05% on both X-ray and CT images; however, the approach suffers from data overfitting issues. Ge et al. (45) presented a multi-label CXR disease diagnosis approach using illness and health label dependencies. The model was comprised of two distinct sub-CNNs that were trained using pairs of different loss functions, i.e., binary cross-entropy, multi-label softmax loss, and correlation loss. The authors further introduced bilinear pooling to compute meaningful features for fine-grained categorization. This method (45) exhibits an AUC of 0.8398 using ResNet as base model; however, it suffers from high computational complexity.

The studies described above have shown remarkable outcomes; however, they are limited to the identification of a few chest-related diseases and lack generalizability for the classification of multiple chest illnesses. A review of approaches for recognizing chest diseases from the literature is given in Table 1. It can be seen that there is still potential for improvement in performing multi-label chest disease classification in terms of accuracy, computation complexity, and generalization ability.

**Table 1:** A comparison of the multi-class chest disease diagnosis.

**Reference**	**Methodology**	**Findings**	**Gaps identified**
Ayan and Ünver (27)	VGG16 Xception network	Accuracy = 0.87% (VGG16) Accuracy = 0.82% (Xception network)	The accuracy can be improved by combining features from both networks
Bhandary et al. (28)	Modified AlexNet (MAN) and Haralick and Hu approach	Accuracy = 97.27%	The generalization performance of the model can be enhanced
Tataru et al. (30)	GoogLeNet, InceptionNet, and ResNet	Accuracy = 80%, F1 score of 0.66	The performance can be improved by the inclusion of a segmentation approach to allow the network to learn more disease-specific attributes
Rajpurkar et al. (31)	Novel CNN (121-layer)	F1 score = 43.5% and AUROC = 0.801	Performance requires further improvement
Albahli (32)	Novel CNN	Accuracy = 87%	Performance needs improvement
Ho and Gwak (33)	A hybrid model with a DenseNet-121 network and hand-crafted feature extractor i.e., SIFT, HOG, LBP, GIST, and different ML classifiers such as SVM, KNN, AdaBoost, and others	Accuracy = 0.8462, F1-score = 0.9413, AUC = 0.8097	Requires improvement in the generalization ability of the model
Abiyev and Ma'aita (34)	Novel CNN	Accuracy = 92.4%	The model can be made deeper to enhance performance
Xu et al. (35)	Densenet169 with multi-scale attention network	AUROC = 0.850	The performance can be improved further
		AUROC = 0.815	
Ma et al. (36)	Densenet121 and densenet169 with cross attention	AUROC = 0.817	The model is computationally complex
		AUROC = 0.775	
Wang and Xia (37)	ResNet-152 with attention network	AUC = 0.781	The model is computationally complex and suffers from high inference time
Ouyang et al. (38)	ResNet with a hierarchical visual attention mechanism	AUC = 0.819	The model is dependent on the availability of box annotations
		AUC = 0.9166	
Pan et al. (39)	DenseNet and MobileNetV2	AUROC = 0.924	The generalizability of the model requires improvement
		AUROC = 0.900	
Albahli and Yar (40)	ResNet50, NasNetLarge, Xception, InceptionV3, and InceptionResNetV2	AUC = 96.9, Sensitivity = 93.4, Specificity = 93.72	The images were segmented before the classification
Alqudah et al. (41)	Novel CNN with softmax classifier, SVM, and KNN	Accuracy = 94%, Sensitivity = 93.33%, Specificity = 96.68%	Performed classification between Normal vs. Bacterial Pneumonia vs. Viral Pneumonia classes
Kim et al. (42)	EfficientNetv2	Accuracy = 82.15%, Sensitivity = 81.40%, Specificity = 91.65%	The evaluation was performed on 4 classes only Pneumonia, Pneumothorax, Tuberculosis, and Normal class
Baltruschat et al. (43)	ResNet38, ResNet50, ResNet101	AUC = 0.822	The performance can be improved further
Ibrahim et al. (44)	CustomVGG19, ResNet152V2, ResNet152V2-GRU, and ResNet152V2-BiGRU	Accuracy = 98.05%, Recall = 98.05%, Specificity = 99.5%, F1-score = 98.24%, AUC = 99.66%	The model is evaluated only using COVID-19, Pneumonia, Lung Cancer, and Normal classes
Ge et al. (45)	ResNet and DenseNet with novel multi-loss function	AUC = 0.8398 (ResNet) AUC = 0.8392 (DenseNet)	The model is evaluated using only four classes

## Proposed methodology

**Figure 1:**
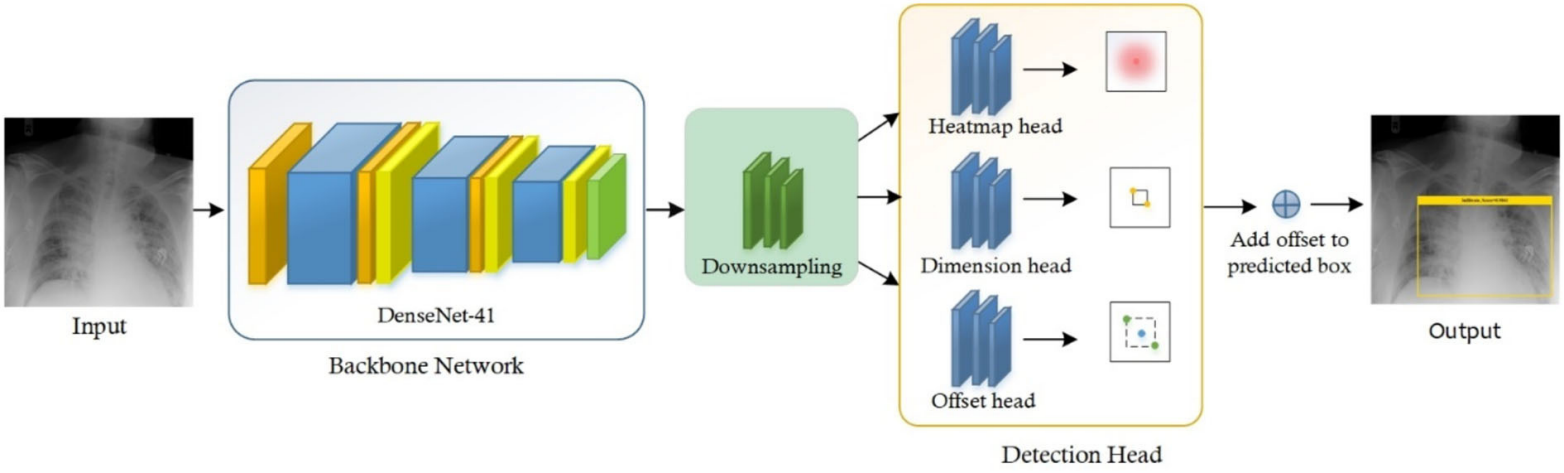
Flow diagram of proposed method namely AI CenterNet CXR.

Chest X-ray disease detection is based on two essential modules: the first is the Localization of chest disease pathologies, and the other is a classification of chest disease into eight categories. The complete functionality of our novel method is described in Figure 1.

For the classification of Chest X-ray disease, we have presented the novel method named CenterNet with Densenet-41. For training of our model, we have the publicly available dataset having eight classes and also their bounding boxes values of disease pathologies. So, we can perform localization of chest X-ray disease lesions directly from images due to the availability of bounding box ground truths. The proposed CenterNet method recognizes the region of interest (ROI) in feature extraction using DenseNet-41, afterward the localized areas are classified into eight classes of chest diseases. Moreover, we have evaluated all samples as per parameters in the field of CV.

### CenterNet

Feature extraction is an essential step for recognizing the regions in images and also for classification. So, efficient features are required to correctly locate the disease areas from CXR images and recognize their categories into eight classes. However, this task is challenging due to the overfitting problem which occurs because of the large feature vector. Another challenge is the skip of essential areas (such as texture, shape, and color changes) of the model due to the small set of the feature vector.

To accomplish the robust and efficient feature vector, it is essential to apply an automated key points extraction approach, avoiding the handcrafted feature methods. Because the handcrafted approaches of features extraction are not effective in correctly recognizing the disease lesions from the CXR images due to different variations, positions, and textures of lesions. To tackle all these problems, we have presented an efficient and novel method, which is the DL method and based on CenterNet. The presented approach named Efficient CenterNet has the ability to directly extract the features efficiently from CXR images. CenterNet has the convolution filter (CF) for key points calculation that extracts the structure of disease areas from images. The inspiration for using the one-stage method i.e., CenterNet (26) over the other object detectors e.g., RCNNs (28) and (15, 29) for chest disease identification is that these are complex structures and take more time due to the two-stage approach. Faster-RCNN uses Region Proposal Network (RPN) for localization of objects from images, then collective features intimate with each ROIs split detection heads and detect the class of object with bounding box. However, these approaches are economically not robust and are not applicable to real-world requirements of object localization. The DL approach CenterNet addresses the issues of the abovementioned methods by identifying features and also the location of ROIs in input parallelly. Moreover, the one-step technique is the ability of CenterNet that makes it more accurate and timelier efficient.

For recognizing and categorizing CXR diseases, it is challenging to locate the features of ROIs because of numerous factors i.e., finding the actual location of ROIs due to extreme color and light variations, and other is finding the category of each object. CenterNet can precisely classify and detect the disease areas of numerous categories through heatmaps, which switched the two-stage into a one-step object detector. The heatmap unit acts by utilizing the center features that accomplish greater recall values, which facilitate to decrease in the computation cost of feature extraction.

### Customize centernet

**Figure 2:**
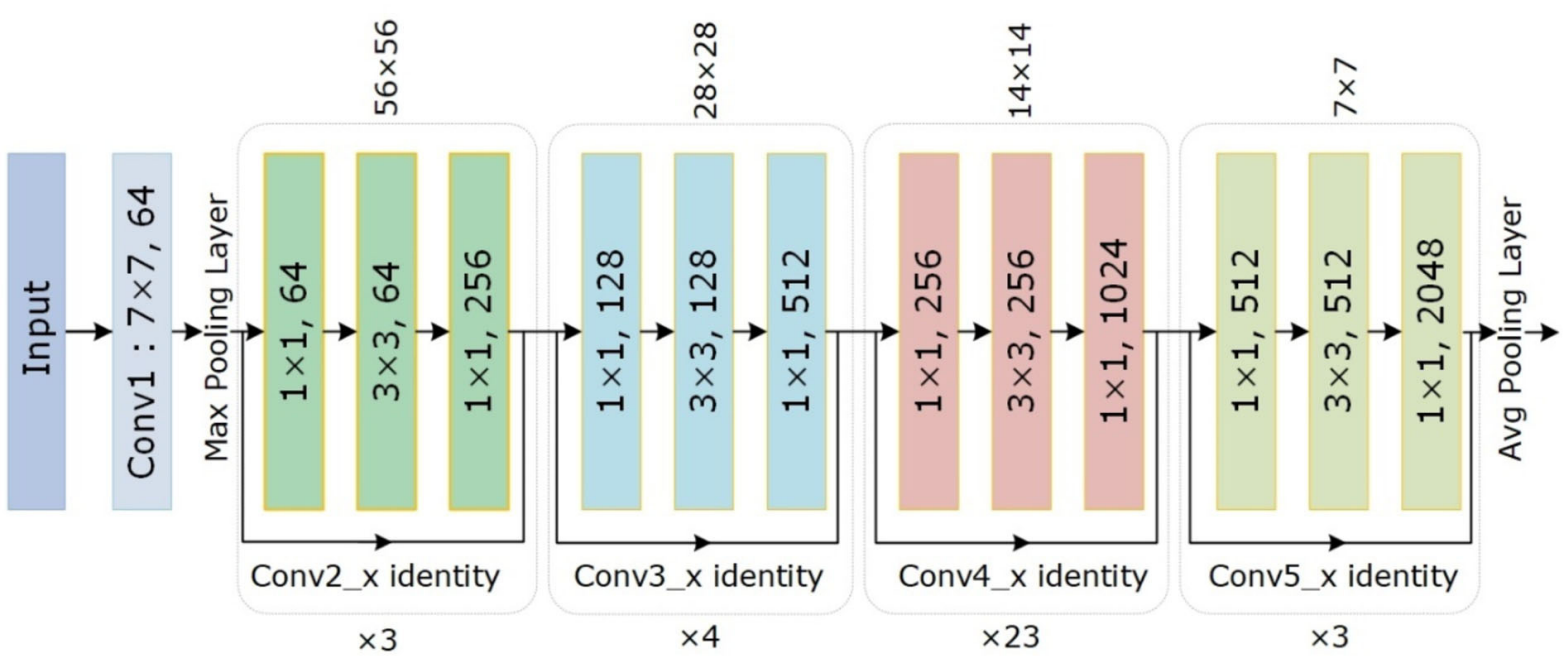
The architectural view of ResNet-101.

The conventional CenterNet (30) used ResNet-101 for computing features to execute medical image analysis. However, this method i.e., ResNet employs skip connections to prevent non-linear conversions, which reason the immediate gradient flow from the previous to the next layers through the identity module. Figure 2 describes the Res-Net-101 technique that encompasses huge parameters and ultimately produces the vanishing gradient problem. To overcome the above issue, we proposed a DenseNet-41 for feature extraction that is densely accompanying the convolution approach. In the presented approach, DenseNet is utilized as a backbone network of CenterNet, which makes CenterNet more efficient due to a smaller number of parameters than ResNet-101. The introduced network consists of numerous Dense Blocks (D_B), which consecutively join up by additional convolutional and pooling layers among successive D_Bs. The DenseNet can exhibit the complex renovation that facilitates overwhelming the challenge of the inadequacy of the output position information for the upper-level key points, in some measure. Moreover, this method encourages feature reproduction, which makes them highly convenient for Chest X-ray disease localization and improves the training procedure. So, we introduced the DenseNet-41 (31) in CenterNet approach for feature extraction from Chest Xray Images.

#### DenseNet-41 feature extractor

**Figure 3:**
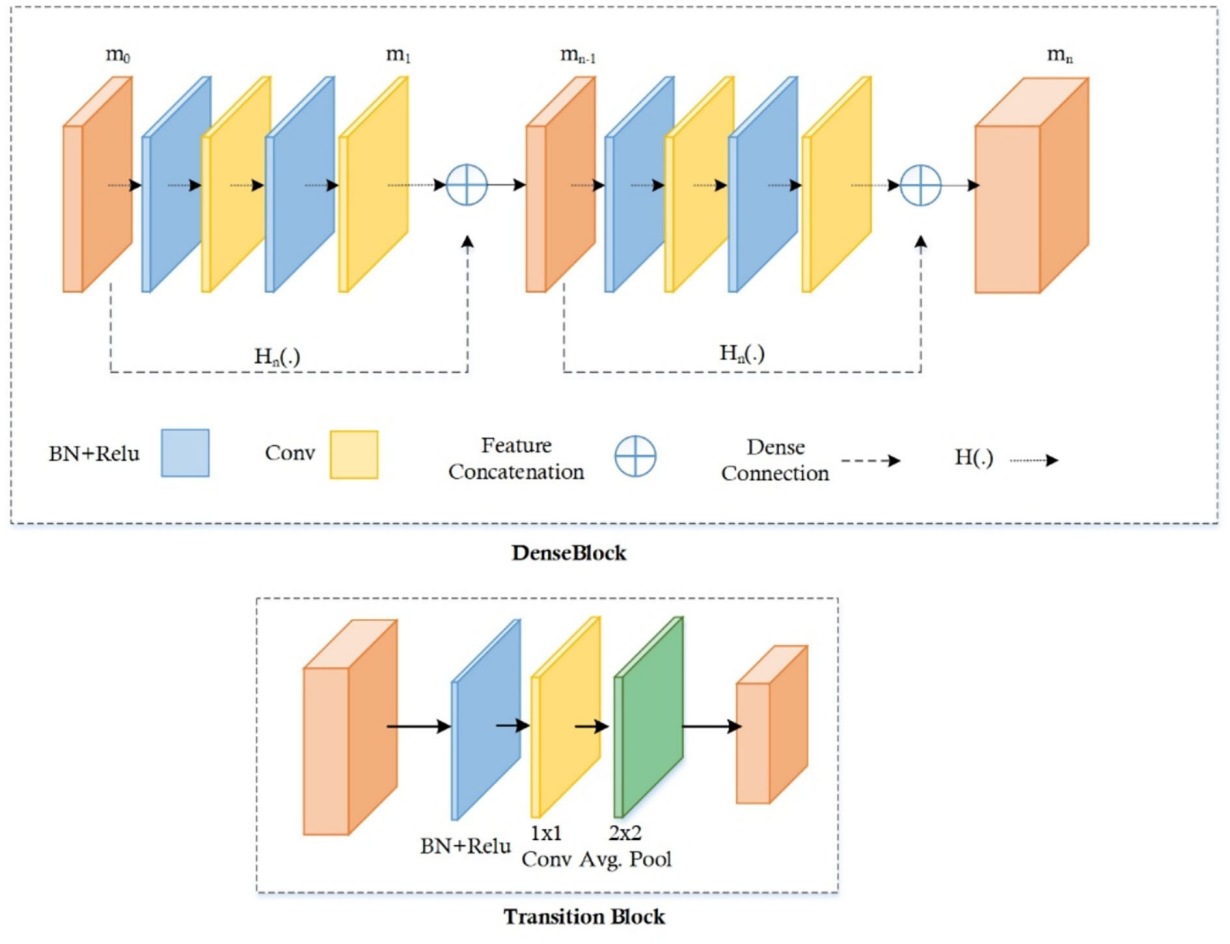
Dense block and transition layer.

DenseNet-41 encompasses four D_Bs along with the equal layers as employed in ResNet-101. The DenseNet-41 has less no of parameters than Resnet-101, which makes it computationally efficient for feature computation of disease detection. Table 2 has the description of DenseNet-41, including D_Bs (as shown in Figure 3), convolutional, and transition layers (T_L).

**Table 2:** Description of DenseNet-41.

**Layer**		**DenseNet-41**	
		**Size**	**Stride**
Con L1		7 × 7 *conv*	2
Pool L1		3 × 3 max_*pool*	2
Dense B1		$\left[\begin{array}{cc} 1\times 1 & conv\\ 3\times 3 & conv \end{array}\right]\times 3$	1
Transition L1	Con L2	1 × 1 *conv*	1
	Pool L2	2 × 2 *avg*_*pool*	2
Dense B2		$\left[\begin{array}{cc} 1\times 1 & conv\\ 3\times 3 & conv \end{array}\right]\times 6$	1
Transition L2	Con L3	1 × 1 *conv*	1
	Pool L3	2 × 2 *avg*_*pool*	2
Dense B3		$\left[\begin{array}{cc} 1\times 1 & conv\\ 3\times 3 & conv \end{array}\right]\times 6$	1
Transition L3	Con L4	1 × 1 *conv*	1
	Pool L4	2 × 2 *avg*_*pool*	2
Dense B4		$\left[\begin{array}{cc} 1\times 1 & conv\\ 3\times 3 & conv \end{array}\right]\times 3$	1
Classification Layer		7 × 7 *avg*_*pool*	
		FC layer	
		SoftMax	

The D_B is the vital component of DenseNet, l × l × m0 demonstrates the key points maps (KM) of the *L*-1 layer. N specifies the dimension of KM, whereas all channels are characterized by m0. P(.) is the non-linear conversion that contains different modules i.e., batch normalization (BaN), ReLU activation method, a 1 × 1 Conv layer (C_L), utilized to lessen all the channels, and a 3 × 3 C_L, used for features reorganization. Dense links are represented by long-dashed arrows, which are utilized to join the *L*-1 to the *L* layer and combined them through the result of the P(.). Lastly, l × l × (m0 + 2m) is the result of the L + 1 layer.

The numerous dense connections enhance KMs; so, the T_L is activated for reduction in feature size from the previous DB, which is briefly explained in (32, 33). The calculated key points are down-sampled with the four stride rate, after that these features are utilized for the estimation of various heads, illustrated in the proceeding subsections.

FMs increase because of vast dense links, so the T_L is represented to decrease the size of the feature map from the preceding D_B (32, 33). The feature set comes from the DenseNet-41 is put down using four stride rate and then transfer to calculate the several heads which are explained below:

#### Heatmap head

This head offers a key points approximation on the reduced deep key points from the DenseNet-41 to find the diseased portions with their category. The respective features are the center of bbox when localize the ROIs can be calculated as follows:


(1) $$\begin{array}{r} {\hat{O}}_{i,j,c}=\exp\left({-}^{{\left(i-{\hat{p}}_{i}\right)}^{2}+{\left(j-{\hat{p}}_{j}\right)}^{2}}{/}_{2\sigma_{p}^{2}}\right) \end{array}$$

where *i* and *j* are the original feature values, ${\hat{p}}_{i}$ and ${\hat{p}}_{j}$ are the positions of estimated down-sampled features, σ_*p*_ displays the region size-adaptive standard deviation, *c* is the total of categories, and *o*_*x*_,_*y*_,_*c*_ shows the center for a candidate features, in case it is marked as 1 means affected; or else, considered as healthy.

#### Dimension head

This type of head is utilized for the prediction of values of bbox, which is responsible for computing the dimensions of the box. The width and height of the bbox can be computed by the L1 norm i.e., (*x*_2_-*x*_1_, *y*_2_-*y*_1_), for the k object with values (*x*_1_, *x*_2_, *y*_1_, *y*_2_).

#### Offset head

After applying down-sampling on input images, the discretization error appears that needs to be minimized. So, the offset head is calculated for this purpose and then the center points are again represented in the high-resolution input image.

#### Multitask loss

Multitask loss is the technique to improve the performance of DL-based approaches like CenterNet, our proposed technique used this type of loss for performance enhancement with accurate localization and classification of disease regions. So, the multitask loss is represented with L on every head, which can be estimated as follows:


(2) $$\begin{array}{r} L_{centernet}=L_{map}+{\text{λ}}_{\dim}L_{\dim}+{\text{λ}}_{off}L_{off} \end{array}$$

The total loss calculated by our method is *L*_*CenterNet*_, in which heatmaps, offset, and dimension head losses are described by *L*_*map*_, *L*_dim_, and *L*_*off*_, respectively. And λ_dim_ and λ_*off*_ are equal to constant values of 0.1 and 1 simultaneously.

The *L*_*map*_ is calculated through the following equation:


(3) $$\begin{array}{r} L_{map}=\frac{-1}{n}\sum_{i,j,c}\{\begin{array}{l} {\left(1-{\hat{\text{O}}}_{i,j,c}\right)}^{\alpha }\log({\hat{\text{O}}}_{i,j,c})\;if\;{\hat{\text{O}}}_{i,j,c}=1\\ {\left(1-{\text{O}}_{i,j,c}\right)}^{\beta }{\left({\hat{\text{O}}}_{i,j,c}\right)}^{\alpha }\log(1-{\hat{\text{O}}}_{i,j,c})\;otherwise \end{array} \end{array}$$

The total key points are shown by *n*. *O*_*i,j,c*_ is the center of the original feature center, whereas **o**_*i,j,c*_ is the estimated value of the center. Hypermeters of loss in our case is described by α and β having the values of 2 and 4 for the whole test.

The *L*_dim_ can be estimated by using the Equation 4,


(4) $$\begin{array}{r} L_{\dim}=\frac{1}{n}\overset{n}{\underset{k=1}{\sum }}|{\hat{b}}_{k}-b_{k}| \end{array}$$

where *b*_*k*_ is the actual and ${\hat{b}}_{k}$ is the predicted bbox coordinates, total samples are shown by *n*.

Ultimately, the *L*_*off*_ is determined by the Equation 5:


(5) Loff=1n∑p|F^p^−(pR−p^)|

The predicted offset rate is denoted by ${\hat{F}}_{\;}$, while R is the resultant stride. The real key point is *p*, while $\hat{p}$ is the down-sampled value.

####  Creation of bounding box

Lastly, the estimated values with each category are processed separately which are gained through heatmaps. In this work, we have utilized the 8 nearest neighbors, and then the highest 100 values are considered.

Let $\hat{Q\;}$ is producing *N*-related center points of class *c* using Equation 6:


(6) $$\begin{array}{r} Q={\left\{\left({\hat{x}}_{j},{ŷ}_{j}\right)\right\}}_{j=1}^{N} \end{array}$$

where the location of every estimated point is symbolized as (${\hat{x}}_{j},{ŷ}_{j}$). We have utilized all values of key points denoted by $\hat{Q\;}$, and bbox and coordinates can be found through Equation 7:


(7) $$\begin{array}{l} ({\hat{x}}_{j}+\partial {\hat{x}}_{j}-{\hat{w}}_{j}/2,\;{\hat{y}}_{j}+\partial {\hat{y}}_{j}-{\hat{h}}_{j}/2,\\ {\hat{x}}_{j}+\partial {\hat{x}}_{j}+{\hat{w}}_{j}/2,\;{\hat{y}}_{j}+\partial {\hat{y}}_{j}+{\hat{h}}_{j}/2) \end{array}$$

In Equation 7, $\left(\partial {\hat{x}}_{j},\partial {\hat{y}}_{j}\right)$ = offset prediction, while (${\hat{w}}_{j},{\hat{h}}_{j}$) = size prediction.

The final bbox is created immediately from the valuation of the features with no usage of IoU-based non-maxima suppression.

### Detection process

CenterNet is an efficient technique as compared to other methods, which are explained in previous sections. So, in this method, input X-ray image along their bbox is given to the trained framework, whereas the CenterNet estimates its center values of disease regions. The complete flow of the introduced solution is described in Algorithm 1.

**Table:**
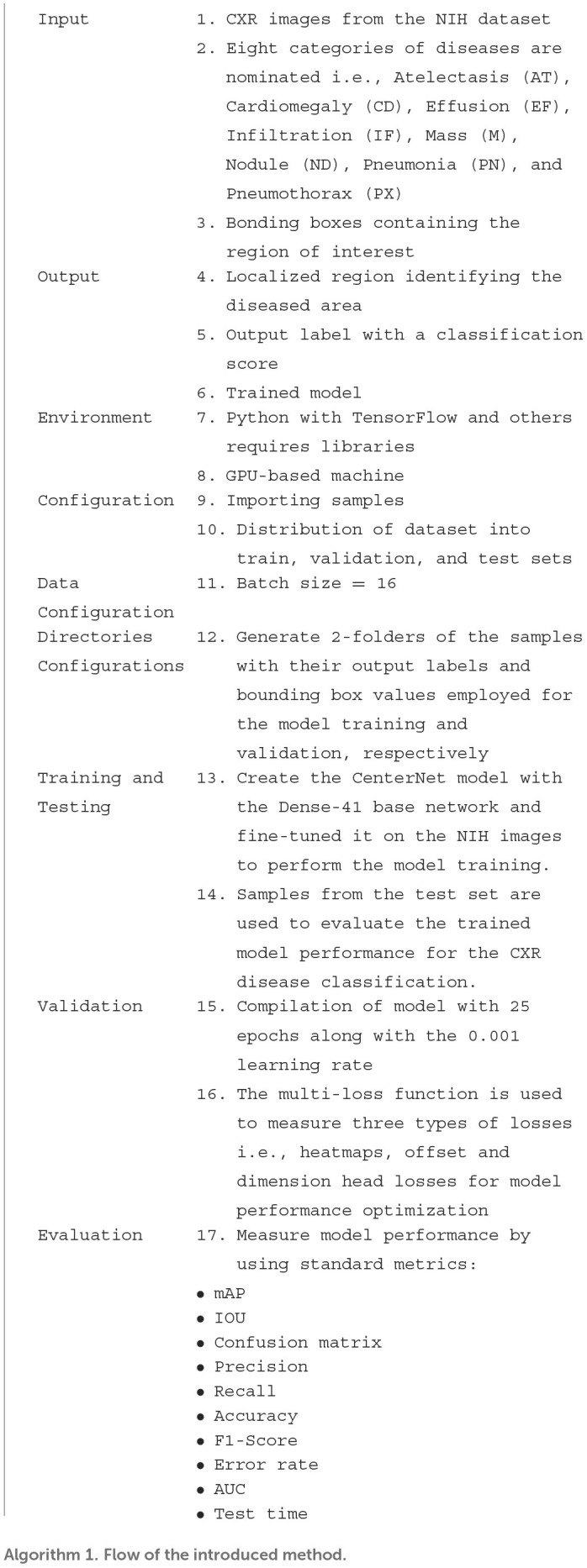


## Experiment and results

In this portion of the paper, we have provided detailed information about the dataset being used for the model verification. Further, we have elaborated on the evaluation measures that are used to compute the quantitative results of our approach. Besides, extensive experiments have been performed to test the proposed approach in numerous ways to show its robustness for CXR disease detection and classification. We have performed the experiments in Python language by using an Nvidia GTX1070 GPU-based system. In the presented technique for CXR recognition, the CenterNet model is employed with pre-trained weights obtained from the MS-COCO dataset, and transfer learning is carried out on the NIH X-ray dataset to modify it for the chest disease classification.

### Dataset

**Figure 4:**
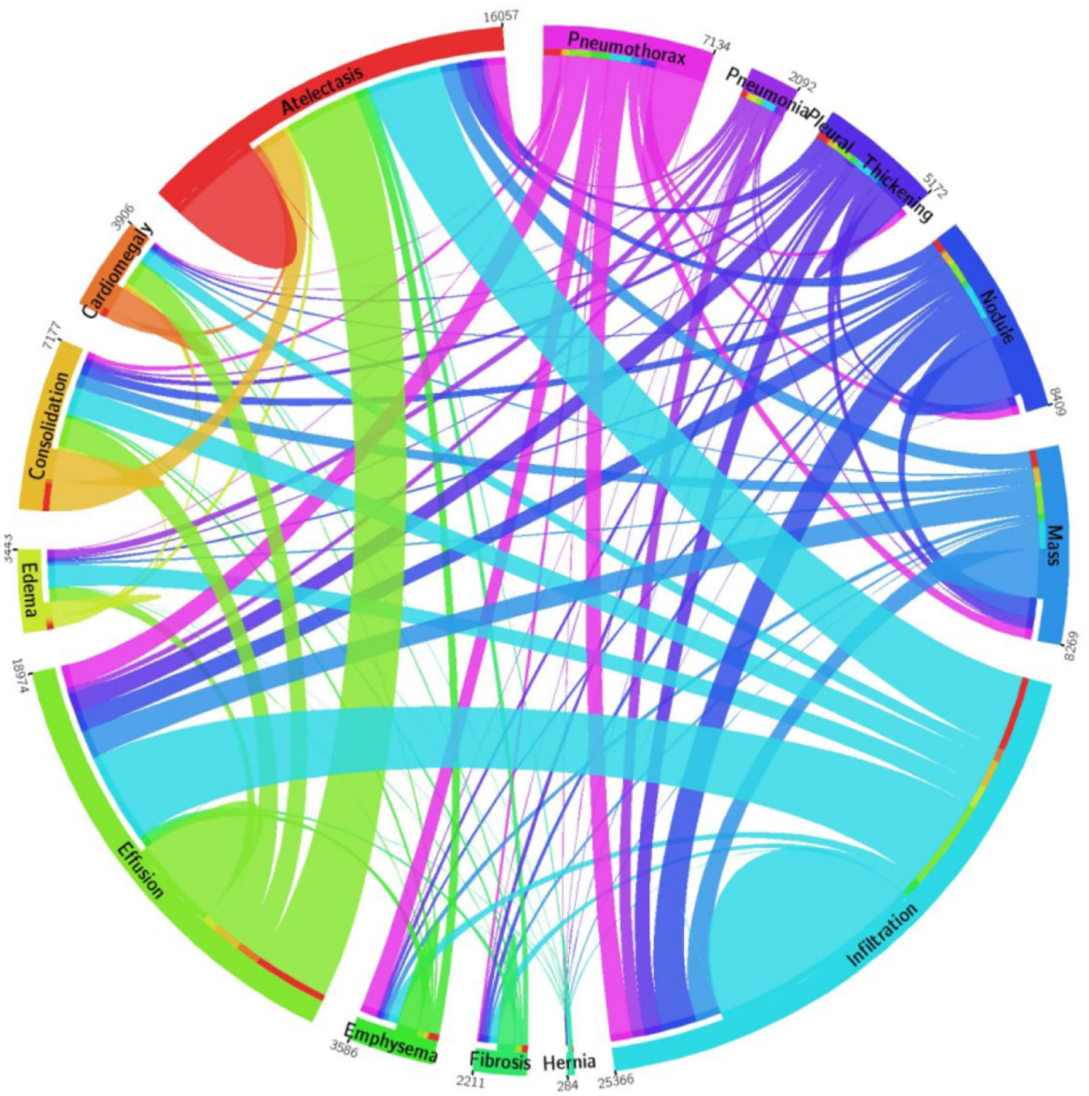
A pictorial view of sample information from the NIH Chest X-ray dataset (47).

**Figure 5:**
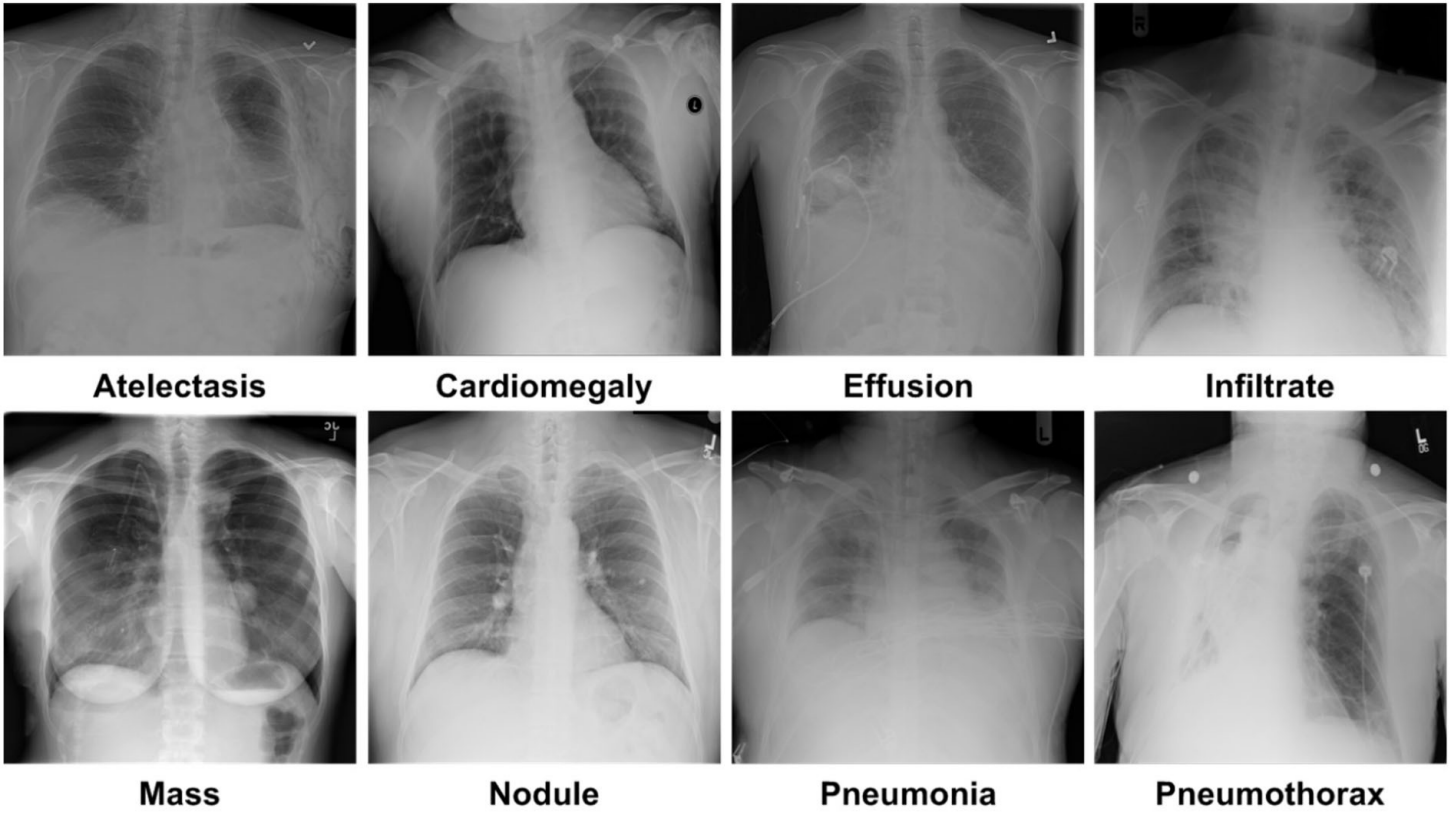
Samples images of NIH CXR dataset.

For model training and testing, we have used a standard dataset of CXR namely the NIH Chest X-ray dataset (46). The employed database comprises a total of 112,120 samples from 30,805 subjects. The details about the entire NIH CXR dataset are shown in Figure 4. The outer layer in the figure shows the number of images in the respective class, and the second outer layer represents all the 14 classes. The complete dataset has 14 classes, however, the dataset contains the annotations for eight types of chest diseases such as AT, CD, EF, IF, M, ND, PN, and PX, respectively. There are a total of 984 annotated samples available for model training, which are marked by a panel of radiologists. As the proposed work is concerned with the employment of an object detection-based model for the CXR classification, therefore, we have considered the abovementioned eight diseases for our approach. A few samples from the NIH CXR dataset are presented in Figure 5. The used dataset is quite complex in nature due to the presence of intense light variations, noise, blurring, color changes, and class imbalance problems.

### Performance metrics

To assess the CXR detection and classification performance of the proposed Custom-CenterNet model, we have utilized several standard metrics used in the area of object detection and classification domain. We have used the mean average precision (mAP), Intersection over Union (IOU), precision, accuracy, and recall, metrics for performance analysis. The mathematical description of the accuracy measure is given in Equation 8:


(8) $$\begin{array}{r} Accuracy=\frac{TP+TN}{TP+FP+TN+FN} \end{array}$$

Equation 9 depicts the mathematical formulation of AP, and equation 10 is the mAP measure, where *AP* shows the average precision for all classes and t is the test sample. *T* is representing all test samples:


(9) $$\begin{array}{r} AP=\overset{1}{\underset{0}{\int }}p\left(r\right)dr \end{array}$$

Here, p(r) is the accuracy of the target area or detection:


(10) $$\begin{array}{r} mAP:=\overset{T}{\underset{i=1}{\sum }}AP\left(t_{i}\right)/T \end{array}$$

**Figure 6:**
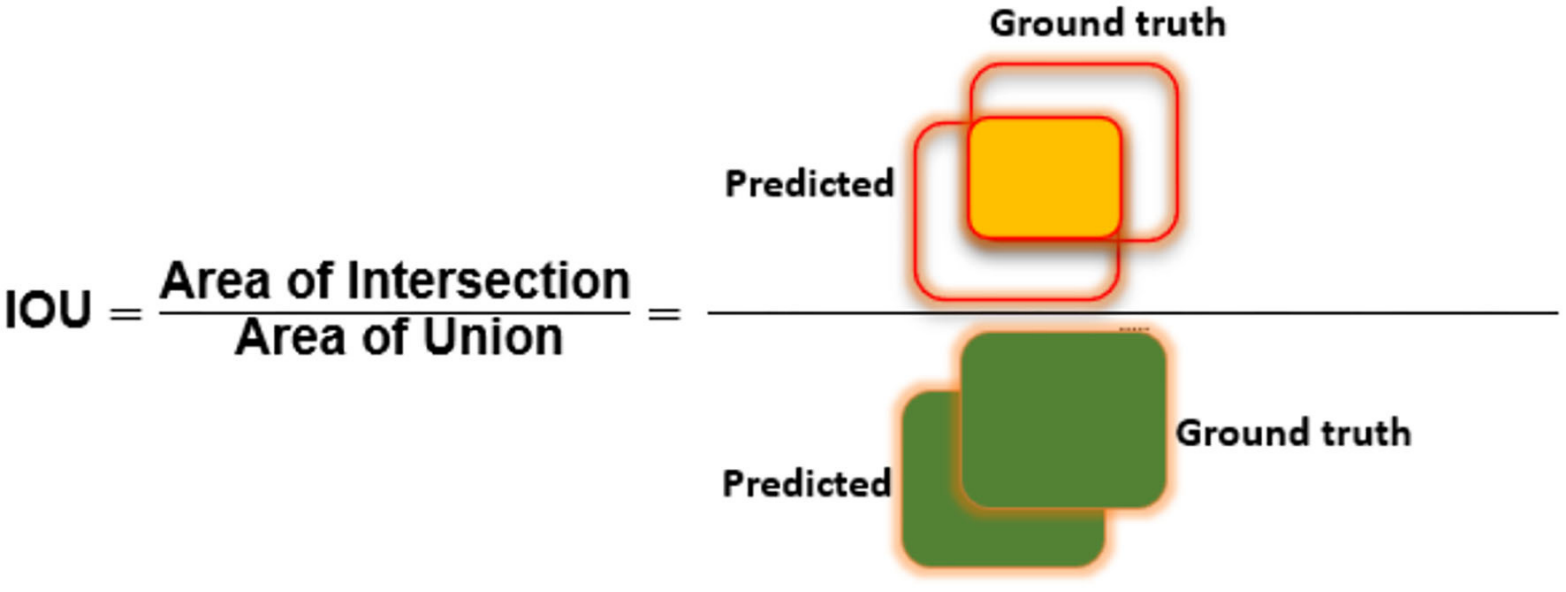
Visual depiction of IOU metric.

**Figure 7:**
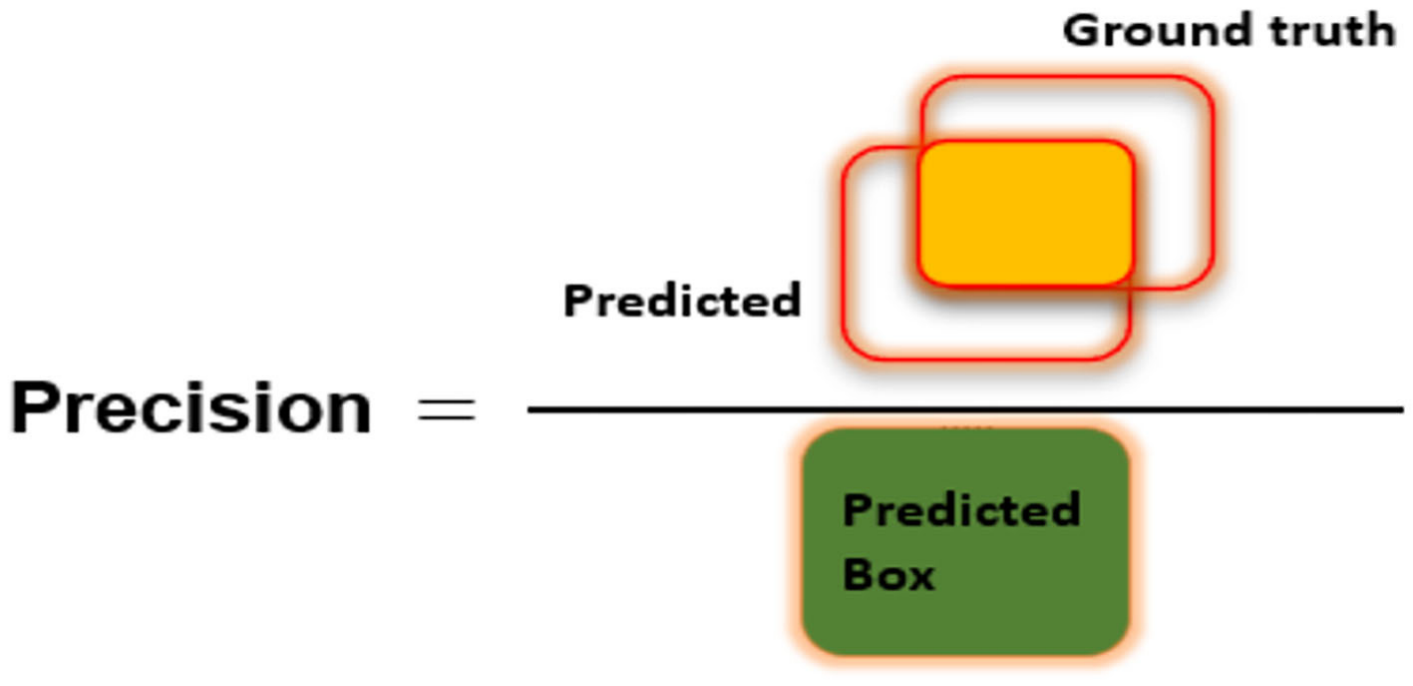
Visual demonstration of Precision metric.

**Figure 8:**
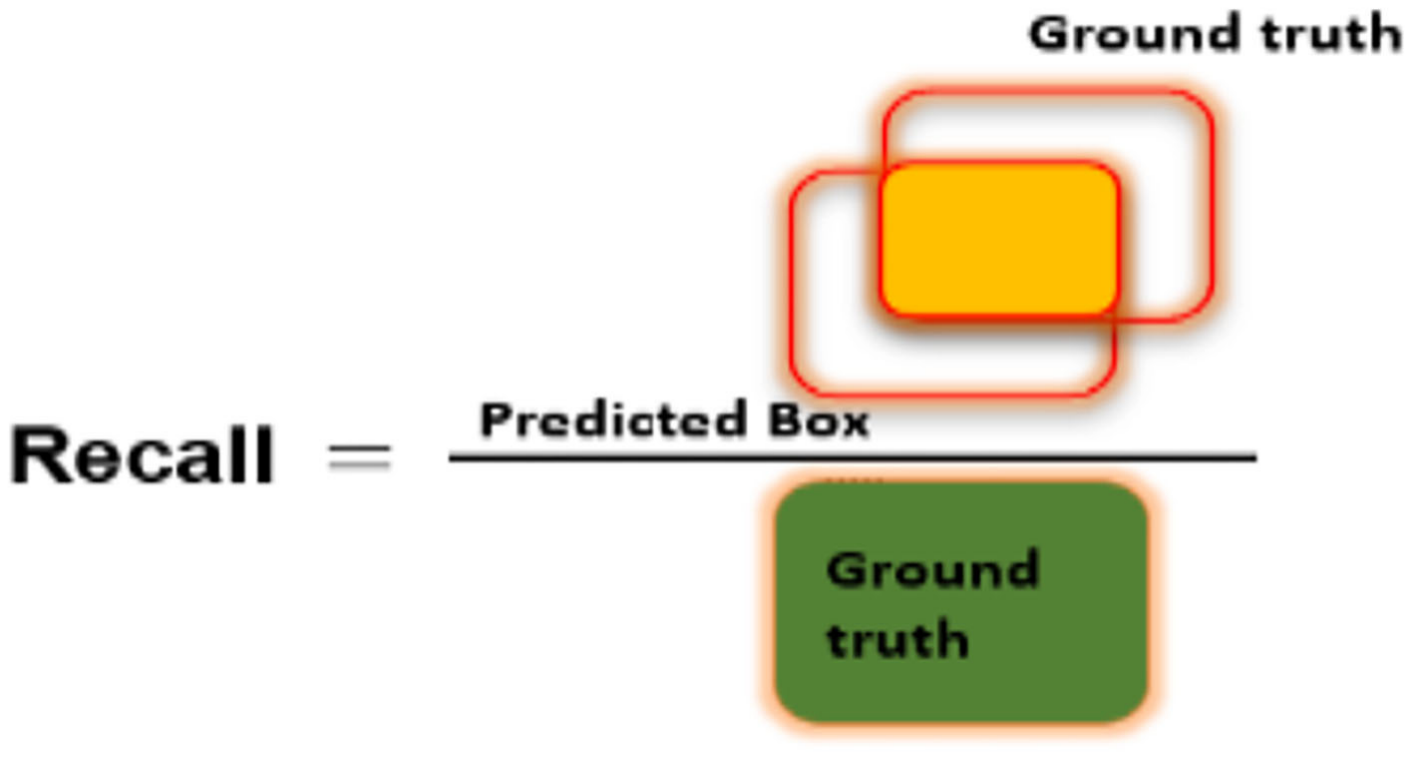
Pictorial representation of Recall measure.

Figures 6–8 explain the visual demonstration form of IOU, precision, and recall, respectively.

### Localization results

**Figure 9:**
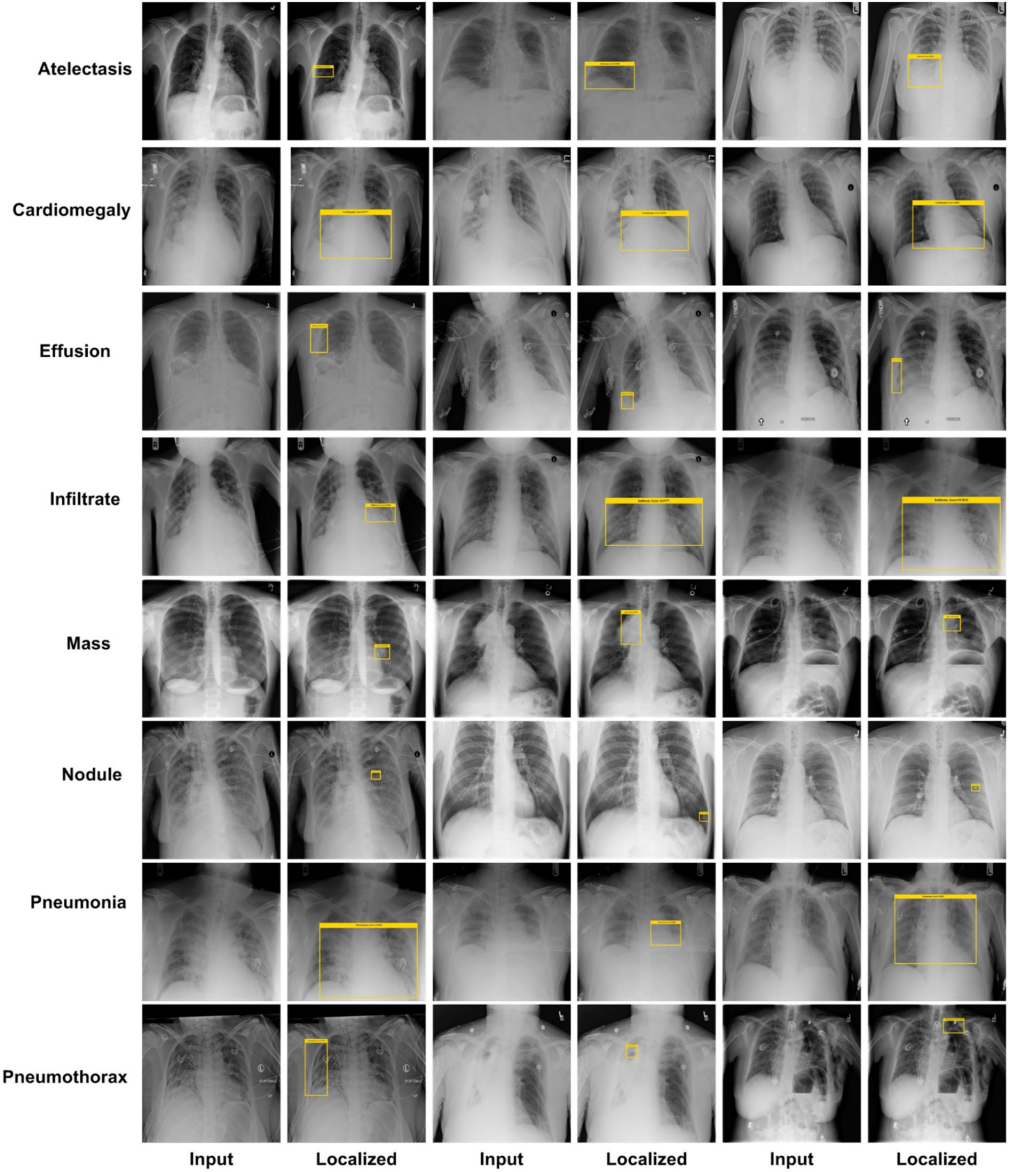
Localization results of the proposed method.

An effective CXR disease classification should be capable of correctly recognizing and classifying all categories of diseases. For this reason, we have performed an analysis to check the CXR abnormalities detection and classification performance of our approach. The test images from the NIH CXR dataset are applied to confirm the localization and categorization power of the custom CenterNet approach, and visual samples are reported in Figure 9. We have reported some test results in Figure 9 for all eight classes, which include class labels and confidence scores. The first row is showing the localization results of the Atelectasis class, the second row is for the Cardiomegaly class. Similarly, the remaining six rows in Figure 9 show the detection results for Effusion, Infiltration, Mass, Nodule, Pneumonia, and Pneumothorax, respectively. From localization results, we analyze that this dataset has both smaller and larger disease regions such as Effusion and Nodule diseases have smaller affected areas, while others have larger affected regions. So, our model can detect both the smaller and larger regions precisely with better results. The samples shown in Figure 9 having different intensity variations are depicting that our model can accurately identify the diseased portion and can differentiate several chest diseases efficiently. Moreover, the model is capable of reliably locating the diseased portion for the distorted samples, which are depicting the robustness of our method. For example, in Figure 9, the second case of the last row has a smaller region and is also similar to the background area, but our method detected it accurately. To numerically discuss the localization ability of the DenseNet41-based CenterNet approach, we have computed the mAP score which is the standard evaluation metric and we have acquired the mAP score of 0.91. From both the visual and quantitative results analysis, we can say that the proposed custom CenterNet model can be reliably applied for the CXR disease identification and classification.

### Class-wise results

Here, we have elaborated the class-wise results of our approach to elaborate the recognition power of our approach in categorizing eight types of chest diseases from the X-ray image modality. For this reason, we employed the DenseNet41-based CenterNet framework on all the suspected samples from the NIH CXR database and computed the performance in the forms of precision, recall, accuracy, and F1 measure.

**Figure 10:**
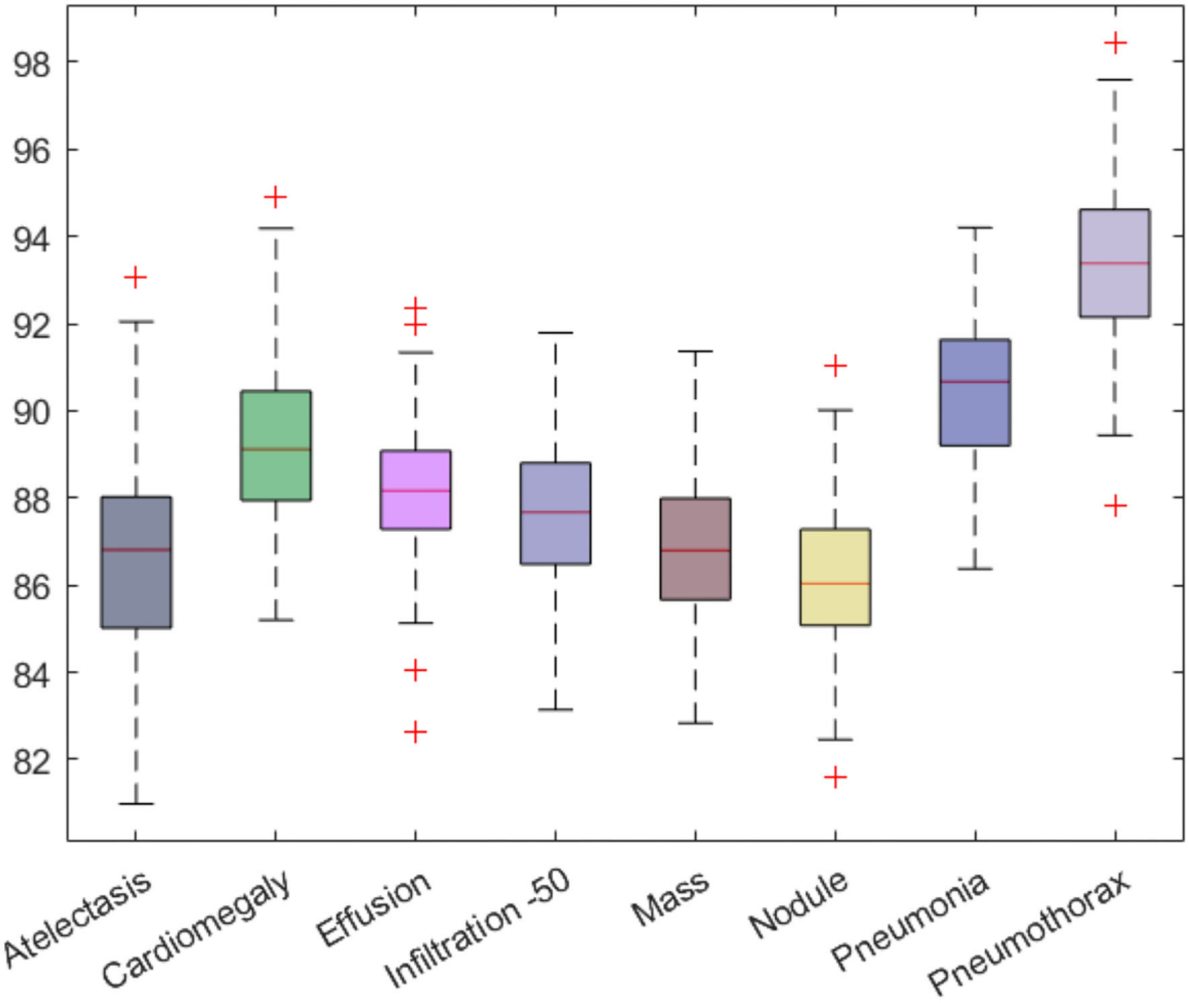
Class-wise precision values for the custom CenterNet model.

Firstly, we have reported the category-wise obtained precision values for our approach as this metric permits us to check how much a model is competent in discriminating the diseased images from the normal samples. The acquired results are shown in Figure 10 from where it is quite visible that our approach has correctly detected the affected samples. More clearly, we have obtained the average precision value of 89%, which is showing the efficacy of the presented technique.

**Figure 11:**
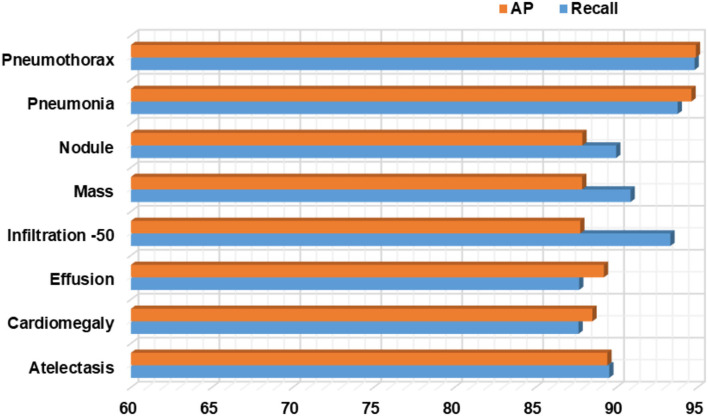
Class-wise AP and recall values for the proposed Custom CenterNet approach.

Moreover, we have computed the recall evaluation metric as it allows us to analyze how much a framework is capable of differentiating the different diseases from each other. The obtained AP and recall values are shown in Figure 11, which is clearly showing that our proposed model is empowered to correctly recognize all eight types of CXR abnormalities and shows an average recall value of 91%.

**Figure 12:**
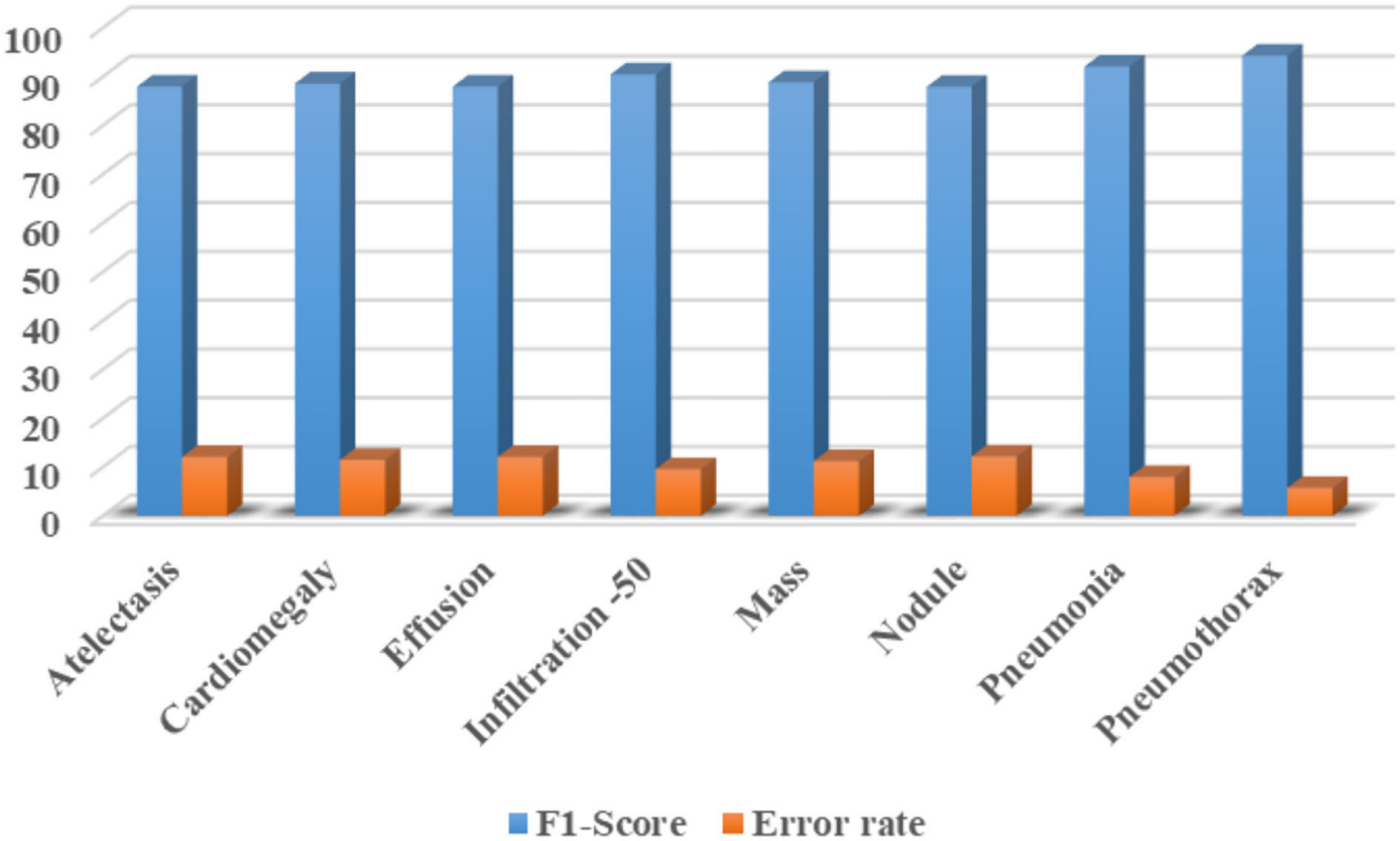
Class-wise F1-Score along with the error rate for CXR diseases classification using custom CenterNet model.

Furthermore, we have computed the F1-Score as the more the value of the F1-Score the better the model performance. The calculated F1-Score along with the error rate for all eight classes of CXR abnormalities are shown in Figure 12. The custom CenterNet approach shows the maximum F1 score of 94.30% along with the minimum error rate of 5.70% for the Pneumothorax class while reporting the lowest F1-Score of 87.88% along with a maximum error rate of 12.12% for the Nodule abnormality. More clearly, we have attained the average F1-Score and error rate of 89.99 and 10.01%, respectively.

**Figure 13:**
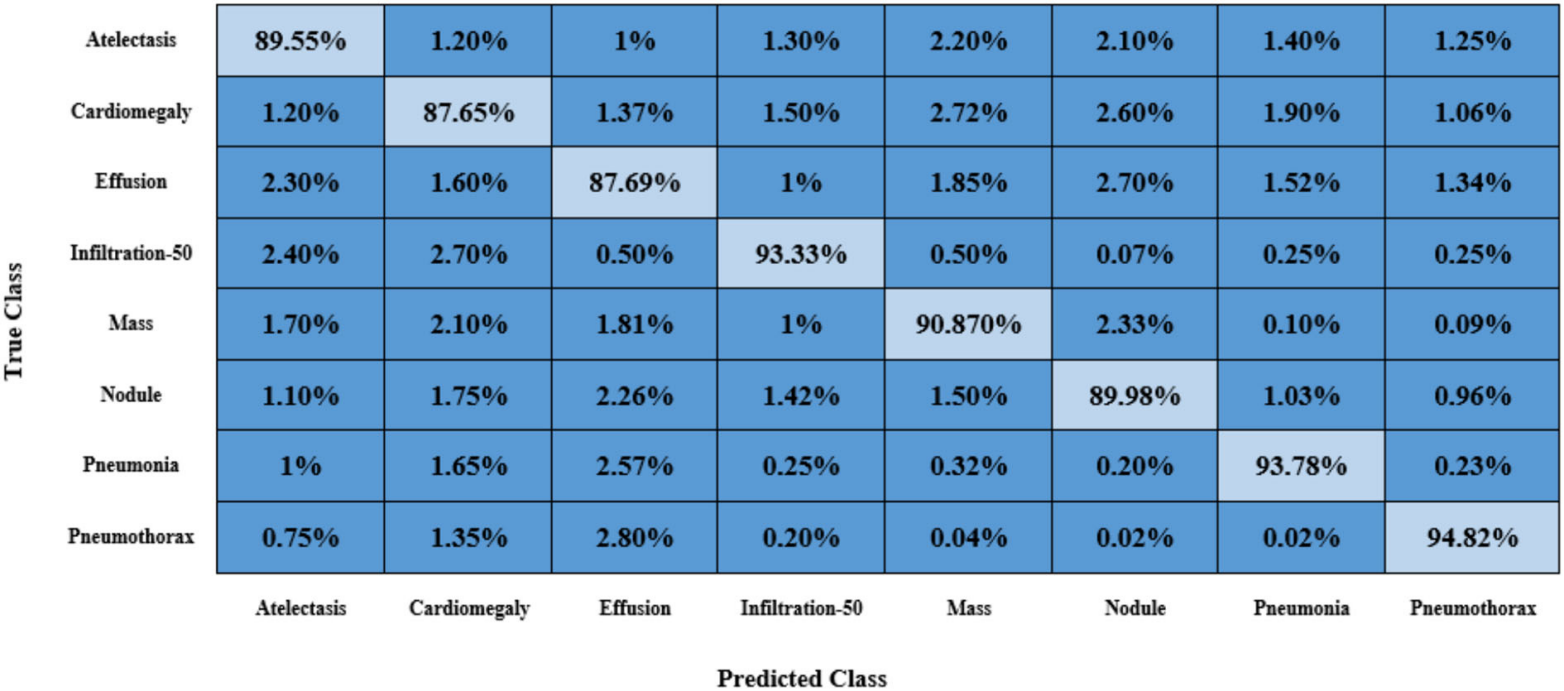
Confusion matrix obtained for CXR disease classification with the custom CenterNet.

Furthermore, we have reported the confusion matrix to further demonstrate the CXR abnormality categorization power of the proposed approach as the confusion matrix is capable of showing the classification performance of a model in a viable manner by showing the actual and predicted values. More descriptively, we have acquired the True Positive Rate (TPR) of 89.55, 87.65, 87.69, 93.33, 90.87, 89.98, 93.78, and 94.82%. It is quite evident from Figure 13 that the presented method can efficiently discriminate the affected regions of several classes of CXR diseases.

**Figure 14:**
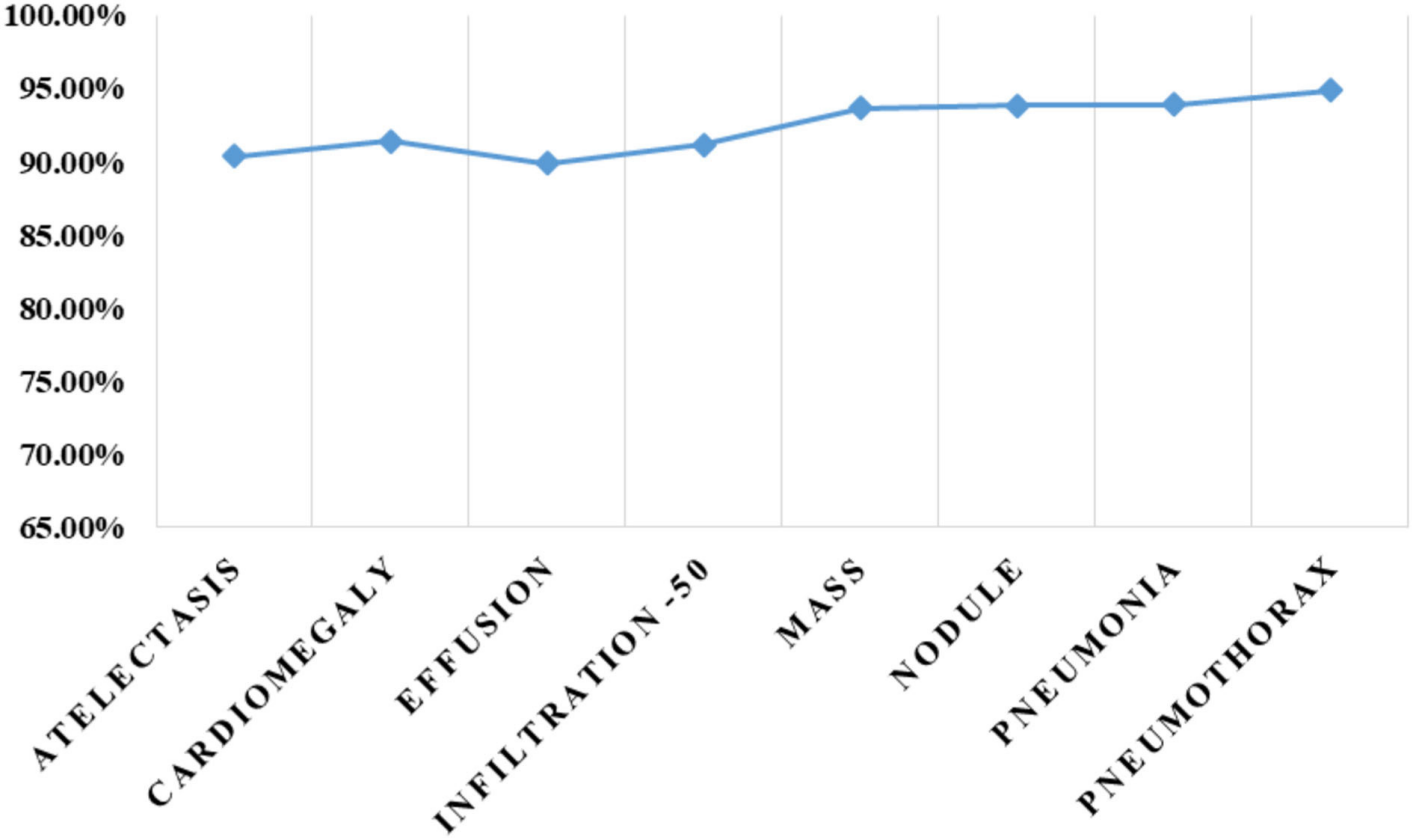
Class-wise accuracy values.

Finally, we have calculated the accuracy values for all eight classes of the CXR diseases, and values are shown in Figure 14 from where it is quite evident that the proposed approach shows robust classification results for all classes. More clearly, we have acquired an average accuracy value of 92.21%. Based on the conducted analysis, we can say that our approach shows better classification performance in terms of all performance measures due to its efficient feature computation power.

### Evaluation of proposed model

In this section, we have provided a comparison of the classification results of our approach against other DL-based methods. For this reason, we have selected the AlexNet (48), GoogleNet (49), VGG16 (50), and ResNet50 (51) models by considering their results for the CXR disease classification as mentioned in (52).

Initially, we performed the class-wise performance analysis of our approach with the nominated DL approaches, and the results are elaborated in Table 3. It can be seen from the table that the DenseNet41-based CenterNet model has outperformed the other approaches for all categories of diseases. More clearly, for the AT and CD diseases, the selected DL methods show the average values of 0.65 and 0.73 which are 0.88 and 0.95 for our case. So, for the AT and CD diseases classification, we have shown performance gains of 22.75 and 22.25%, respectively. While for the EF, IF, and M chest diseases, we have given the average values of 0.78, 0.91, and 0.85, while the comparative methods show the average values of 0.68, 0.60, and 0.54, respectively, so we have shown the performance gains of 24.5, 17.75, and 36.75% for the mentioned diseases, respectively. Similarly, for the ND, PN, and PX chest diseases, the peer approaches report the average values of 0.645, 0.57, and 0.735, which are 0.85, 0.84, and 0.96 for the proposed approach. Hence, we have presented the 20.5, 27, and 22.5% of performance gains for the ND, PN, and PX chest disease classification, respectively. Entirely, for all diseases, the competent methods attain the average AUC value of 0.645, while our work acquires 0.887, hence we have provided an overall performance gain of 24.20%.

**Table 3:** Comparison with base models in terms of the AUC metric.

**Model**	**Atelectasis**	**Cardiomegaly**	**Effusion**	**Infiltrate**	**Mass**	**Nodule**	**Pneumonia**	**Pneumothorax**
AlexNet	0.64	0.69	0.66	0.60	0.56	0.65	0.55	0.74
GoogLeNet	0.630	0.70	0.69	0.61	0.54	0.56	0.59	0.78
VGG16	0.63	0.71	0.65	0.59	0.51	0.65	0.51	0.63
ResNet50	0.71	0.81	0.74	0.61	**0.56**	0.72	0.63	0.79
Proposed	**0.88**	**0.95**	**0.93**	**0.78**	**0.91**	**0.85**	**0.84**	**0.96**

The bold means highest AUC metric.

In the second phase, we assessed the custom CenterNet approach with the nominated DL approaches by comparing the results on the entire dataset using several standard metrics, namely, precision, recall, accuracy, and F1-measure. The comparative analysis is shown in Table 4 from where it is quite clear that the proposed framework is more efficient for CXR abnormality categorization. We have obtained the highest performance values for all the evaluation measures with the values of 89, 91, 92.21, and 89.99% for the precision, recall, accuracy, and F1-Score, respectively. The second largest results are shown by the EfficientNet with the values 87.74, 88.95, 88.01, and 87.61% for the precision, recall, accuracy, and F1-Score respectively. DenseNet-121 attained better results, however, this model is computationally complex as compared to our proposed DenseNet-41. Furthermore, the ResNet50 model the values of 77, 75, 77.63, and 75.99% for the precision, recall, accuracy, and F1-Score, respectively. Moreover, the AlexNet model shows the lowest classification results with values of 65, 66.14, 67.45, and 65.57% for the precision, recall, accuracy, and F1-Score, respectively. From the conducted analysis, we can say that the proposed DenseNet41-based CenterNet model is quite efficient to recognize each category of chest diseases and show robust performance on the entire dataset as compared to the other DL-based approaches. The main cause for the enhanced classification results of our model is because of the usage of the DenseNet41 as its base network, as this model employs the shallow network architecture which permits it to select a more reliable set of images key points. While comparatively, the selected DL-based approaches are quite complex in structure and unable to perform well for the samples with intense light, and color variations causes decrease in their performance for the CXR abnormalities recognition. So, we can say that our model presents an efficient and effective solution for classifying chest disease from the X-ray image modality.

**Table 4:** Comparative comparison with base models.

**Model**	**Precision**	**Recall**	**Accuracy**	**F1-Score**
AlexNet	65.00%	66.14%	67.45%	65.57%
GoogLeNet	69.53%	71.88%	70.35%	70.69%
VGG16	72.00%	74.32%	75.41%	73.14%
ResNet-50	77.00%	75.00%	77.63%	75.99%
Inception V4	79.32%	75.65%	79.32%	79.22%
DenseNet-121	83.01%	81.84%	83.21%	82.87%
EfficientNet	87.74%	88.95%	88.01%	87.61%
Proposed	89.00%	91.00%	92.21%	89.99%

### Comparison with other object detection models

Here, we experimented to analyze the results of our approach by comparing it against several other DL-based object recognition approaches for the CXR abnormality categorization. For this reason, we have taken both the one and two-stage techniques. The major distinction between the one and two-stage object detection models is that in the case of two-stage approaches, initially numerous region proposals are created to identify the location of the diseased portion, and then the associated class is determined. While for the one-stage object detection methods, the position and class of RoI are determined in a single step. In the case of two-stage approaches, we have chosen the Fast-RCNN (53), Faster-RCNN (4, 54), and Mask-RCNN (55) models, while for the other, we have taken the RetinaNet (56) and conventional CenterNet (21) models.

For performance comparison, we have used the mAP performance measure as it is the highly designated metric used in the area of object recognition. Additionally, the test time of all competitor methods is also considered to discuss the computational efficiency as well. The obtained comparison is shown in Table 5 from which it is quite evident that our approach is proficient for CXR disease classification both in terms of performance results and test time with the values of 0.91 and 0.21 s, respectively. The Fast-RCNN model employs the hardcoded-based approaches for its key points computation that are unable to tackle the image distortions reliably. The Faster-RCNN and Mask-RCNN approaches have tackled the issues of the Fast-RCNN model; however, these are computationally inefficient due to their two-stage networks. Whereas, the RetinaNet approach is unable to learn the discriminative anchors for the acentric key points of suspected samples. We also compared our model with the YOLO object detector, it achieved a 0.76 mAP value and the test time is 0.22 s. This model is faster, however, attained a low localization rate because it strives to detect small regions of disease from the images.

**Table 5:** Comparison with object detection models.

**Model**	**Base**	**mAP**	**Test time (sec/img)**
Fast-RCNN	VGG-16	0.65	0.28
Faster-RCNN	VGG-16	0.77	0.25
Mask-RCNN	ResNet-101	0.79	0.23
RetinaNet	ResNet-101	0.63	0.27
YOLO	ResNet-50	0.76	0.22
CenterNet	ResNet-101	0.82	0.25
**Proposed CenterNet**	DenseNet-41	0.91	0.21

The conventional CenterNet model shows better performance; however, still unable to generalize to real-world scenarios due to its high computational cost. The proposed approach that is the DenseNet41-based model has better addressed the limitations of existing approaches by identifying the diseased portion in a more viable manner. The major cause for the better performance of our model is due to the employment of the DenseNet41 model as a feature extractor, which empowers it to better designate the image features which in turn enhances its recognition power and reduces its time complexity as well.

### Comparative analysis against ML classifiers

We have further explained the robustness of our approach for the CXR disease recognition by evaluating its results against the Conventional ML-based classifiers. For this reason, we have nominated two renowned ML classifiers named the SVM and KNN, and obtained values are shown in Table 6. The values in Table are clearly showing that the presented approach obtains the highest AUC with the value of 0.887. The second highest result is attained by the SVM classifier with the value of 0.745, while the KNN classifier shows the lowest value of 0.721, respectively. More descriptively, the comparative classifiers show the average value of 0.733, which is 0.887 for the proposed work. So, we have given a performance of 15.40%. The comparative analysis is clearly depicting that the presented custom CenterNet is more proficient in classifying the several diseases of the chest from the X-ray image modality because of its high recognition ability.

**Table 6:** Comparison with ML-based classifiers.

**Classifier**	**AUC**
SVM (57)	0.745
KNN (57)	0.721
**Proposed**	**0.887**

The bold means highest AUC metric.

### Comparative analysis with state-of-the-art methods

In this part, a comparative analysis is executed in comparison to several latest approaches introduced for the CXR disease classification employing the same dataset. For a fair comparison, the highest average results reported in (52, 58–62) are taken and evaluated against our obtained average results.

Initially, we have compared the proposed approach in terms of the AUC metric and the obtained comparison is reported in Table 7. Wang et al. (58) proposed a DL-based approach for the CXR disease classification, where the CNN-RNN framework was introduced to compute the deep features from the input samples and perform the classification task. The work (58) acquired an average AUC value of 0.753. Another DL-based approach was presented in (59) employing the concept of boosted cascaded convents and attained the average AUC value of 0.778. Liu et al. (60) introduced an approach namely the Contrast-Induced Attention Network (CIA-Net) that used the concept of constructive learning to perform the CXR abnormalities recognition and show the average AUC value of 0.801. Seyyed-Kalantari et al. (61) presented a CNN-based approach to categorize several diseases of the chest via employing the X-ray modality and obtained the average AUC value of 0.821. Han et al. (62) presented a residual-based approach for recognizing several CXR diseases and acquired an average AUC value of 0.838. While in comparison, the presented approach acquired the highest value of the AUC measure with the value of 0.837. More descriptively, for the AT disease, the competent approaches show an average value of 0.786 and 0.880 in our work; hence, we presented a performance gain of 9.40%. For the CD, EF, and IN classes, the competitor methods show the average values of 0.894, 0.856, and 0.698, respectively, which are 0.99, 0.93, and 0.95 for our technique. Therefore, for the CD, EF, and IN classes, the custom CenterNet approach shows the average performance gains of 9.6, 7.4, and 15.2%, respectively. Similarly, for the M, ND, PN, and PX classes, the presented framework provides the average performance gains of 10.2, 10.8, 9.6, and 0.4%, respectively. While collectively, the approaches in (58–62) show the average AUC value of 0.789, while our method shows the average AUC value of 0.888 and presented the performance gain of 8.98%, which is showing the robustness of our approach for the CXR abnormalities classification.

**Table 7:** Comparison of latest approaches in terms of the AUC metric.

**Approach**	**Atelectasis**	**Cardiomegaly**	**Effusion**	**Infiltrate**	**Mass**	**Nodule**	**Pneumonia**	**Pneumothorax**
Wang et al. (58)	0.73	0.84	0.79	0.67	0.73	0.69	0.72	0.85
Kumar et al. (59)	0.76	0.91	0.86	0.69	0.75	0.67	0.72	0.86
Liu et al. (60)	0.79	0.87	0.88	0.69	0.81	0.73	0.75	0.89
Seyyed-Kalantari et al. (61)	0.81	0.92	0.87	0.72	0.83	0.78	0.76	0.88
Han et al. (62)	0.84	0.93	0.88	0.72	0.87	0.79	0.77	**0.90**
**Proposed**	**0.88**	**0.99**	**0.93**	**0.95**	**0.90**	**0.84**	**0.84**	0.88

The bold means highest AUC metric.

Secondly, the performance comparison of our work in terms of IOU is discussed against the latest methods reported in (52), and obtained comparison is presented in Table 8. Wang et al. (52) introduced a deep CNN model for identifying and classifying the CXR diseases and attained the average IOU value of 0.569. Similarly, a CNN-based approach was introduced in (62) and acquired an average IOU value of 0.746. Li et al. (63) proposed a Residual-based approach for classifying the CXR abnormalities and attained an average IOU value of 0.728. In comparison, our proposed custom CenterNet model exhibits the average IOU value of 0.801 which is the greatest among all peer methods. More clearly, the peer techniques show the average IOU value of 0.681 which is 0.801 for the proposed solution. Hence, for the IOU measure, the custom CenterNet model gives the average performance gain of 12%.

**Table 8:** Comparison of latest techniques in terms of the IOU metric.

**Approach**	**Atelectasis**	**Cardiomegaly**	**Effusion**	**Infiltrate**	**Mass**	**Nodule**	**Pneumonia**	**Pneumothorax**
Wang et al. (52)	0.69	0.94	0.66	0.71	0.40	0.14	0.63	0.38
Han et al. (62)	0.72	0.96	0.88	0.93	0.74	0.45	0.65	0.64
Li et al. (63)	0.71	0.98	0.87	0.92	0.71	0.40	0.60	0.63
**Proposed**	**0.76**	**0.99**	**0.93**	**0.95**	**0.74**	**0.56**	**0.74**	**0.74**

The bold means highest IOU metric.

From the conducted analysis, it is quite clear that the proposed approach for the CXR disease classification is more competent in terms of both IOU and AUC evaluation measures as compared to the latest approaches. The major reason for the robust recognition power of the proposed solution is due to the more discriminative feature computation ability of our model, which assists it to recognize all categories of disease in an efficient manner. While in comparison, the approaches in (52, 58–62) are quite complex in structure which results in the model over-fitting issue. Moreover, the approaches are unable to deal with several distortions of suspected samples such as color and light variations which make them inefficient to capture the image information accurately. While in comparison, our technique is more effective to tackle the transformation changes in the suspected samples. Hence, we can say that the presented custom CenterNet is more competent for CXR disease recognition and categorization.

## Conclusion

In our work, we presented AI-CenterNet CXR, an end-to-end DL-based framework for the automated recognition and categorization of thoracic illness from chest radiographs. Our method is based on a CenterNet model that uses the DenseNet network for the computation of effective image attributes. More specifically, we integrated the DenseNet-41 network to extract a discriminative set of key points from the chest x-rays for the accurate identification of abnormalities. Moreover, due to the one-stage object detector framework CenterNet model, the suggested architecture is computationally robust to classify several CXR abnormalities. We conducted extensive experiments using the NIH CXR dataset to show the effectiveness of the proposed approach. Our technique attained an overall AUC of 0.888, an average precision value of 89%, a recall value of 91%, and an IOU of 0.801 to identify and classify eight categories of chest illness. According to the results, the proposed technique outperforms existing approaches in terms of both time and computational complexity. Moreover, the approach can correctly identify the aberrant regions and categorize the various types of chest illness in the presence of distortions, significant inter-class similarities, and intra-class variances. In the future, we will incorporate fourteen classes and perform experiments on other latest DL-based models.

## Data availability statement

The original contributions presented in the study are included in the article/supplementary material, further inquiries can be directed to the corresponding author/s.

## Author contributions

SA: conceptualization, methodology, software, and writing-original draft preparation. TN: data curation, writing-original draft preparation, validation, supervision, and writing-reviewing and editing. All authors contributed to the article and approved the submitted version.

## Conflict of interest

The authors declare that the research was conducted in the absence of any commercial or financial relationships that could be construed as a potential conflict of interest.

## Publisher's note

All claims expressed in this article are solely those of the authors and do not necessarily represent those of their affiliated organizations, or those of the publisher, the editors and the reviewers. Any product that may be evaluated in this article, or claim that may be made by its manufacturer, is not guaranteed or endorsed by the publisher.
